# Photoprotective Properties of Extracts Derived from *Neoglaziovia variegata* (Arruda) Mez Incorporated into Mesoporous TiO_2_

**DOI:** 10.1021/acsomega.5c06956

**Published:** 2025-10-27

**Authors:** Francisco A. A. Miranda, Vaeudo V. Oliveira, Roosevelt D. S. Bezerra, Josy A. Osajima, Edson C. Silva-Filho

**Affiliations:** † 119484 State University of Maranhão (UEMA), Campus de Caxias, Caxias, Maranhão 65604-380, Brazil; ‡ State University of Piauí (UESPI), Campus Prof. Barros Araújo, Picos, Piauí 64602-000, Brazil; § Federal Institute of Education, Science and Technology of Piauí (IFPI), Teresina-Central Campus, Teresina, Piauí 64000-040, Brazil; ∥ Interdisciplinary Advanced Materials Laboratory (LIMAV), Materials Science and Engineering Graduate Program, 529937 Federal University of Piaui (UFPI), Teresina, Piauí 64049-550, Brazil

## Abstract

The development of safe and effective photoprotective agents has gained increasing interest in recent years, particularly through the incorporation of natural compounds into inorganic matrices. In this study, sunscreen formulations were developed by incorporating *Neoglaziovia variegata* (Arruda) Mez extracts into mesoporous TiO_2_, using different solvent fractions: ethyl acetate, ethanol, chloroform, and hexane. Structural characterization confirmed the efficient incorporation of the extracts into the mesoporous channels of TiO_2_, without compromising its crystallinity. Chemical analysis showed that the ethanolic and ethyl acetate extracts had the highest total phenolic content (1.550 ± 0.041 and 1.346 ± 0.004 mg GAE g^–1^, respectively) and the highest antioxidant activity (IC_50_ values of 49.03 ± 1.24 and 59.34 ± 1.44 μg mL^–1^, respectively). These results directly correlated with higher Sun Protection Factor (SPF) values. The pure ethyl acetate and ethanol extracts showed the highest SPF values, reaching 26 and 10.5, respectively. Notably, the incorporation of the extracts into TiO_2_ resulted in a marked increase in photoprotective efficacy. For example, the chloroform and hexane extracts, which had isolated SPF values of 3.9 and 1.1, reached values of 35 and 33, respectively, after incorporation, demonstrating a synergistic effect between the inorganic matrix and the plant’s bioactive compounds. Toxicological tests performed on blood agar and *Artemia salina* indicated that the presence of TiO_2_ significantly reduced the toxicity of the pure extracts, demonstrating their biocompatibility. Spreadability tests revealed good coverage capacity for all formulations, favoring easy and uniform skin application. These results suggest that the developed hybrid systems represent a promising and safe alternative for the formulation of natural sunscreen products.

## Graphical abstract

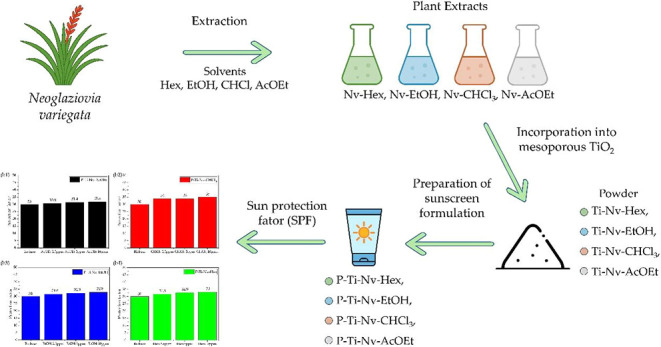

## Introduction

Ultraviolet (UV) radiation presents some benefits for humans, such as improved cardiovascular health, endogenous vitamin production, antibacterial effects, among others. On the other hand, UV radiation poses risks to humans, especially to the skin, potentially causing skin disorders such as erythema, burns, and immunosuppression, in addition to promoting premature skin aging and being responsible for skin cancer due to excessive exposure.^1^

This occurs because the ozone layer protects humans only from UVC radiation (100–280 nm) and is not capable of blocking UVA (320–400 nm) and UVB (280–320 nm) radiation.^2^ UVA radiation is the main responsible for photoaging, as it penetrates deeper into the dermis and damages DNA through the production of reactive oxygen species. UVB radiation is associated with sunburns and the direct damage to DNA through the formation of cyclobutane pyrimidine dimers (CPDs) and pyrimidine-pyrimidone photoproducts. In this context, exposure to UVA and UVB radiation increases the risk of basal cell carcinoma, squamous cell carcinoma, and melanoma.^3^

In this context, the search for materials that can be used as photoprotective agents against UV radiation has intensified in recent years. In this regard, sunscreens have been one of the main forms of protection against the harmful effects of UV radiation.^1–3^ Thus, a good sunscreen should not only provide effective protection against UV radiation damage but also be nontoxic to the ecosystems that may come into contact with its components.^4^

These protectors are generally organic or inorganic synthetic molecules that can absorb, reflect, or disperse UV rays, depending on their chemical composition. Additionally, they must be photostable and nontoxic to human skin cells. These conventional sunscreens have shown negative effects, suggesting that their effectiveness may be limited. Additionally, if they are photounstable, they can induce adverse phenomena in skin cells, contributing to an increase in skin cancer cases. Beyond their direct impact on human health, they can also affect the ecosystem, leading to negative environmental consequences.^5^

In this context, the use of natural agents derived from plants has emerged as a promising alternative for the production of natural sunscreens. These agents demonstrate greater efficiency as photoprotectors and free radical neutralizers, while also exhibiting lower toxicity for both the environment and human health compared to conventional sunscreens.^5^ These natural agents exhibit photoprotective and antioxidant properties due to the presence of secondary metabolites, such as terpenoids, anthocyanins, flavonoids, carotenoids, and phenolic acids, among others, found in plants.^6,7^

Thus, the combination of plant extracts with sunscreens can increase the sun protection factor (SPF), acting as additional filters due to their synergistic effects and, consequently, enhancing the protection these products provide to the skin. In this regard, *Neoglaziovia variegata* (Arruda) Mez, an endemic Bromeliaceae popularly known as ’caroá’, has attracted interest due to its chemical potential. *N. variegata* grows spontaneously in the semiarid region of northeastern Brazil and is often found in the Caatinga, except in its most humid areas.^8–10^ Additionally, the fruit of *N. variegata* is known in folk medicine, where its tea is used to treat cough, bronchitis, flu, and pneumonia. Previous studies have demonstrated that this plant exhibits antimicrobial, antinociceptive, and gastroprotective activities, as well as antioxidant and photoprotective properties, possibly associated with the presence of phenolic compounds and flavonoids.^8–10^ Moreover, studies have shown that extracts derived from *N. variegata* can be used as sunscreens in pharmaceutical formulations, presenting promising results regarding their photoprotective efficacy.^8,9^ The chemical analyses of the plant have revealed the presence of a flavone aglycone and rutin, both of which have antileukemic activity against MOLM-13 cells.^10^

Given the abundance of *N. variegata* in the Brazilian Northeast, its traditional use in folk medicine, and the properties already reported in studies, it is essential to deepen research on the photoprotective potential of its extracts. In this regard, the literature still lacks studies that evaluate the incorporation of these extracts into mesoporous TiO_2_. This combination is of great interest because mesoporous TiO_2_ has gained attention for its unique properties, such as high adsorption capacity, low cost, nontoxicity, large surface area, and photostability. Furthermore, this material is photoactive under visible light, and its photocatalytic activity can be enhanced when combined with other materials, making it a promising candidate for the development of new sunscreen formulations.^11,12^

Considering that there are other plants with higher amounts of phenolics content, the choice of *N. variegata* was made to take advantage the potential of the local flora. Thus, the selection of *N. variegata* for the development of a sunscreen formulation is strongly justified by its phytochemical profile and biological properties. While previous studies have demonstrated various activities of the plant, such as antimicrobial, antinociceptive, and gastroprotective effects, its choice for this work is based primarily on its proven antioxidant and photoprotective properties.^8–10^ These activities are directly linked to the presence of phenolic compounds and flavonoids, which are recognized in the literature for their ability to neutralize free radicals generated by UV radiation and for their effectiveness in absorbing UV wavelengths. Therefore, the rich phenolic content of *N. variegata* positions it as a promising and rational candidate for the creation of a natural photoprotective agent.

Given the potential of *N. variegata* and the properties of mesoporous TiO_2_, the combination of these materials for the development of new photoprotective formulations represents a promising and still unexplored area of research. Considering the absence of studies on this approach in the literature, this study aims to develop sunscreens with photoprotective and antioxidant properties by combining extracts of *N. variegata* with mesoporous TiO_2_ in the anatase phase.

## Materials and Methods

### Plant Material

The leaves of *N. variegata* were collected in the city of Canto do Buriti—in the state of Piauí—Brazil. The samples were identified and deposited by a botanist from the Herbarium Graziela Barroso of the Federal University of Piauí (UFPI), a voucher specimen (TEPB: 31.226). This technology is registered in the National System for the Management of Genetic Heritage and Associated Traditional Knowledge (SISGEN): #A9842E1.

### Reagents

Titanium­(IV) isopropoxide—97% (Sigma-Aldrich), cetyltrimethylammonium bromide (CTAB)—98.0% (NEON), dimethyl sulfoxide (DMSO)—99.9% (Sigma-Aldrich), methanol—99.8% (Dinâmica), ethanol—99.8% (Dinâmica), ethyl acetate—99.8% (Dinâmica), chloroform—98.8% (Impex), and *n*-hexane—99.0% (Impex). All reagents were used in analytical grade without prior purification.

### Procedure for Obtaining the Bioactive Extract

The preparation of bioactive extracts of *N. variegata* followed methodologies described in the literature with adaptations.^10,13–15^ The leaves were dried at a temperature of 310 K and then placed in an oven at 313 K for 5 h for dehydration. Immediately after, the leaves were ground in a knife mill until they turned into powder. Then, 1200 g of powdered *N. variegata* were immersed in 6.0 L of ethyl alcohol. This mixture was kept under mechanical stirring for 72 h at room temperature. Finally, the *N. variegata*-ethanol extractive solution was filtered under reduced pressure. After filtration, the supernatant was divided into two parts. Part I was subjected to vacuum concentration using a rotary evaporator at 318 K. After this process, the crude extract obtained in ethanol was named (Nv-EtOH).

Part II of the supernatant was transferred to a separatory funnel, and a mixture of deionized water and methanol in a 7:3 ratio (H_2_O/MeOH) was added to this solution, resulting in the phase referred to as *N. variegata* hydroalcoholic (Nv-HA). Subsequently, the Nv-HA phase was subjected to liquid–liquid extraction with hexane, chloroform, and ethyl acetate, in increasing order of polarity, yielding the fractions Nv-Hex, Nv-CHCl_3_, and Nv-AcOEt, respectively. After this step, the solutions were decanted, filtered, and the solvents were removed by evaporation under reduced pressure. Subsequently, all fractions underwent a drying process, remaining in an oven at 313 K for 4 h. To ensure complete removal of moisture, the bioactive fractions of Neoglaziovia variegata were subsequently lyophilized.

### Synthesis of Mesoporous TiO_2_

The present synthesis of mesoporous TiO_2_ was performed using the hydrothermal process according to Zi et al.^16^ This synthesis consists of the dissolution of 10.0 mmol of the Cetyltrimethylammonium bromide (CTAB) in 10.0 mL of ethanol. In this synthesis the CTAB is used as template. The mixture was stirred until it became homogeneous. Then, 10.0 mmol of titanium IV isopropoxide (TIP) was added to the reaction system, used as a source of titanium. The reaction system remained under stirring for another 2 h until the complete hydrolysis and condensation of TIP in the ethanolic medium (EtOH). The solid formed was separated by centrifugation using a time of 10 min at 5000 rpm. After separation, the material was dried in an oven at 373 K for 2 h. Then, the CTAB surfactant was removed by calcination at 823 K for 3 h.

### Incorporation of *N. variegata* Extracts into Mesoporous TiO_2_

The loading/incorporation assays of the extracts (Nv-Hex, Nv-CHCl_3_, Nv-EtOH, and Nv-AcOEt) into mesoporous TiO_2_ were performed using the adsorption method.^17^ For this procedure, 200.00 mg of *N. variegata* extracts and 100.00 mg of mesoporous TiO_2_ were dispersed in 40.00 mL of plant extract solution. The suspension containing mesoporous TiO_2_ and N. variegata was kept under constant mechanical stirring (184 rpm) in an incubator shaker for 72 h at 310 K. After this period, the TiO_2_ samples loaded with the extracts (acetate, chloroform, ethanolic, and hexane phases) were separated by centrifugation at 5000 rpm for 10 min.

### Preparation of Sunscreen Formulation

The sunscreens developed in this study were prepared based on methodologies described in the literature, with adaptations.^18–20^ For this purpose, a ready-made industrial base was used, into which the UV filters Uvinul A Plus, Uvinul T 150, and T-Lite SF-S were incorporated. The final formulation contained the following chemical composition: *Theobroma grandiflorum* seed butter, allantoin, ethylhexyl methoxycinnamate, dimethylamine hydroxybenzoyl hexyl benzoate, ethylhexyl triazone, titanium dioxide, bis-ethylhexyloxyphenol methoxyphenyl triazine, methylene bis-benzotriazolyl tetramethylbutylphenol, decyl glucoside, propylene glycol, caprylic/capric triglyceride, cetostearyl alcohol, ceteareth-20, carbomer, triethanolamine, xanthan gum, BHT, tocopherol, Copaifera officinalis resin, VP/eicosene copolymer, cyclomethicone, dimethazone poly­(dimethylsiloxane), glycerin, trimethylsiloxysilicate, disodium EDTA, phenoxyethanol, caprylyl glycol, and water.

Thus, sunscreen formulations based on *N. variegata* extracts incorporated into mesoporous TiO_2_ were prepared in two steps. In the first step, mesoporous TiO_2_ solutions containing the plant’s bioactive extract were prepared using different solvents. For this purpose, 0.1058 mg of each Ti-Nv complex was weighed and separately dissolved in 0.5 mL of solvent: ethyl acetate (Ti-Nv-AcOEt), chloroform (Ti-Nv-CHCl_3_), ethanol (Ti-Nv-EtOH), and hexane (Ti-Nv-Hex). In the second step, each of these solutions was incorporated into 35.0 g of a ready-made cosmetic base (Biobase-30), resulting in four distinct formulations.

### Toxicological Tests

#### Hemolytic Activity on Blood Agar

The hemolytic activity assays were performed in quadruplicate, following the blood agar diffusion methodology (Labclin) described in the literature with adaptations,^21–23^ using a concentration of 1000.00 μg mL^–1^. Poloxamer at the same concentration was used as a positive control, while dimethyl sulfoxide (DMSO), the solvent used for sample dilution, served as the negative control. Sterilized Whatman No. 1 paper discs (7 mm in diameter) were placed on blood agar plates and impregnated with 25.00 μL aliquots of each tested sample. After application, the plates were incubated at 37 °C for 24 h. At the end of this period, the plates were visually examined for the presence of hemolysis halos, which were measured in millimeters around the discs.

#### Toxicity against *Artemia salina*

The test was conducted according to the methodology proposed by Nichols et al.^23–25^ *Artemia salina* eggs were incubated in saline water at a concentration of 12 ppm for 24 h. After this period, the hatched larvae were collected for the bioassay. Seawater was used as the negative control, and 0.5 mL of dimethyl sulfoxide (DMSO) was used as the positive control.

For the assay, triplicate solutions of the samples were prepared at concentrations of 10, 100, 250, 500, and 1000 μg mL^–1^. Ten *A. salina* nauplii were added to each test tube, and the larvae were kept in contact with the samples for 24 h. After this period, the number of surviving larvae was counted. Larvae were considered dead if they remained immobile for more than 10 s after gentle agitation of the tubes containing *N. variegata* extracts in the ethyl acetate, chloroform, ethanolic, and hexane phases.

#### Determination of Total Phenolics Content

The quantification of total phenolic content in the *N. variegata* extract samples, across the different phases studied, was performed by visible region spectroscopy using the Folin–Ciocalteu method with adaptations as described in the literature.^26,27^ For the analyses, methanolic solutions (solution I) of 100.00 mL containing 1000.00 μg mL^–1^ of each extract (Nv-AcOEt, Nv-CHCl_3_, Nv-EtOH, and Nv-Hex) were prepared. Subsequently, a 7.50 mL aliquot of solution I was transferred to a 50.00 mL volumetric flask and the volume was completed with methanol, yielding solution II.

Solution III was prepared using 100.00 μL of solution II, 500.00 μL of Folin–Ciocalteu reagent, and 6.00 mL of distilled water, followed by stirring for 1 min. After this period, 2.00 mL of a 15% Na_2_CO_3_ solution were added, followed by additional stirring for 30 s. The final volume of solution III was adjusted to 10.00 mL with distilled water. After 2 h, the absorbance of the samples was measured at λ = 750 nm using glass cuvettes. Methanol and all reagents, except for the extract, were used as the blank.

The total phenolic content (TPC) was determined by interpolating the absorbance readings on a calibration curve constructed with gallic acid standards (10.00 to 350.00 μg/mL), and the results were expressed as mg of GAE (gallic acid equivalent) per gram of extract. The equation of the calibration curve was *A* = 0.8875*C* – 0.0086, where *C* represents the gallic acid concentration, *A* is the absorbance at λ = 750 nm, and the correlation coefficient was *R* ^2^ = 0.9933. All analyses were performed in triplicate.

#### Antioxidant Activity Test

The antioxidant activity assays were based on the scavenging capacity of DPPH (2,2-diphenyl-1-picrylhydrazyl) free radicals, following methodologies present in the literature with adaptations.^28,29^ A stock solution of the samples at a concentration of 1000 μg mL^–1^ was prepared by dissolving 5 mg of each sample in 5 mL of HPLC-grade methanol. Using a micropipette (100–1000 μL), the stock solution was diluted to obtain the following concentrations: 0.1, 1, 5, 10, 50, 100, 500, and 1000 μg mL^–1^.

In 96-well microplates, 35 μL of each sample (multichannel micropipette 10–300 μL) was transferred and mixed with 215 μL of DPPH solution at 200 μmol L^–1^ (multichannel micropipette 50–250 μL). Absorbance measurements were taken after 30 min of reaction in the absence of light, using a microplate spectrophotometer at the maximum wavelength (λ_max_) of 515 nm. Blank measurements were performed using the tested sample solutions (35 μL) mixed with methanol (215 μL). Quercetin was used as a positive control and evaluated at the same concentrations. All experiments were carried out in triplicate to ensure reproducibility.

The conversion of absorbance values into percentage of antioxidant activity (%AA) was performed using the following eq 1

1 $$\%\mathrm{AA}=\frac{\left({\mathrm{Abs}}_{\mathrm{DPPH}}-\left({\mathrm{Abs}}_{\mathrm{sample}}-{\mathrm{Abs}}_{\mathrm{blank}}\right)\right)\times 100}{{\mathrm{Abs}}_{\mathrm{DPPH}}}$$

Where, Abs_DPPH_ is the initial absorbance of the methanolic DPPH solution, Abs_sample_ is the absorbance of the reaction mixture containing the sample and DPPH; Abs_blank_ is the absorbance of the mixture containing methanol and the sample only. The EC_50_ values were determined by fitting a dose–response curve using a probit model in Prism software.

#### Sun Protection Factor (SPF) Test

This assay allows the evaluation of the photoprotective efficacy of the cosmetic formulations developed in this study. For this purpose, the samples were dissolved in water at a concentration of 1 mg mL^–1^ and analyzed using a spectrophotometer in the range of 290 to 320 nm, with 5 nm intervals.^30–32^ The sun protection factor (SPF) calculation follows the eq 2

2 $$\mathrm{SPF}=\mathrm{CF}\times \sum_{290}^{320}\mathrm{EE}\left(\lambda \right)\times I\left(\lambda \right)\times \mathrm{Abs}\left(\lambda \right)$$

The constant parameters used in the SPF calculation are as follows: CF, a correction factor with a fixed value of 10; EE­(λ), which represents the erythemogenic effect at a given wavelength (λ); *I*(λ), the intensity of solar radiation at the corresponding wavelength; and Abs­(λ), the absorbance of the sunscreen-containing formulation measured at the same wavelength. Furthermore, EE (λ) and I (λ) values were previously calculated by Sayre et al.^32,33^ as described in Table 1.

**Table 1:** Erythemogenic Effect (EE) versus Radiation Intensity (I) According to Wavelength^a^

λ (nm)	EE × *I* (normalized)
290	0.0150
285	0.0817
300	0.2874
305	0.3278
310	0.1864
315	0.0839
320	0.0180

a EE × *I* (normalized) = erythemogenic effect (EE) versus radiation intensity *I*.

#### pH Determination

The analysis of the pH of the synthesized materials is fundamental to evaluate the feasibility of using these cosmetic products in humans, since these materials must have a pH compatible with the pH of the human skin. The determination of the pH of sunscreens (P–Ti–Nv-AcOEt, P–Ti–Nv-EtOH, P–Ti–Nv-CHCl_3_ and P–Ti–Nv-Hex) were carried out using a pHS-3b digital benchtop pH meter, model pH METER, calibrated with the standard buffer solutions determined by the equipment itself. pH measurements were performed in triplicate at a temperature of 298 K.

#### Determination of Spreadability

The spreadability data is fundamental to understand the extent of the coverage area of the sunscreen on the skin. So, a good protector must have an excellent spreadability index.

The measurements of the spreadability index for the sunscreen formulations were carried out in triplicate using equipment consisting of: square glass mold plate with a 1.2 cm diameter central hole where it was placed on a glass support plate positioned on graph paper. Then, the sample was introduced into the hole in the plate and leveled using a spatula. The mobile plate was removed and a glass plate of known weight was placed over the sample. After 1 min, the diameters covered by the sample in opposite positions were read, with the aid of graph paper, and then the average diameter was calculated.

The spreadability index (*E_i_*) was calculated using eq 3 described by^34,35^

3 $$E_{i}=\frac{d^{2}\cdot \pi }{4}$$

Where *E_i_* represents the spreadability of the sample under weight *i*, expressed in square millimeters (mm^2^); *d* is the average diameter obtained from the measurements, in millimeters (mm); and π is taken as 3.14. In addition, the spreadability values as a function of the added weights were determined through 3 measurements, calculating the average between them. Where, (*E_i_*) is the spreadability index in square millimeters and *d* is the average diameter in millimeters spread over the weights of the plates.

#### Characterizations

X-ray diffraction (XRD) patterns of powdered samples were recorded on a Shimadzu diffractometer model XR-D600 A, using Cu–Kα radiation (λ = 1.54184 Å). For XRD analyses 2θ-range from 1.5 to 80° with scanning rate of 2° min^–1^ and voltage/current standard (40 kV/30 mA). The infrared spectra were collected on a Bomem spectrophotometer; model MB-102 using the DRIFT accessory. The samples in powder form, were analyzed in transmittance mode, in the region of 4000–400 cm^–1^, 64 scans and resolution of 4 cm^–1^. Scanning electron microscopy (SEM) images were prepared by dispersing the silica over a carbon tape glued on a stub and then coated with gold. The images of the surface morphology of the materials were collected using the FEI field emission electron source (SEM-EC) equipment, model FEG-250. The thermogravimetric analysis (TG-DTG) allows monitoring the change in the mass of the sample as a function of temperature variation. The analysis (TG-DTG) were performed with SDT Q600 equipment from TA Instruments. Measurements for each sample, were carried out under an argon atmosphere in the heating range from room temperature to 1273 K, heating rate of 20 K·min^–1^ and entrainment flow of 100 mL min^–1^. Mass spectra were obtained using a mass spectrometer (Amazon X, Bruker Daltonis, IT-ESI-MS) with the respective analysis parameters: electro-spray ionization (ESI) source in negative mode in the mass range *m*/*z* 100 to 1500, direct infusion with syringe flow of 5.00 μL·min^–1^, capillary voltage of 4.0 KV, nitrogen nebulizer with a flow of 5.00 μL·min^–1^ at a pressure of 8 psi and source temperature of 320 K.

## Results and Discussion

### Characterizations

Figure 1 presents the X-ray diffraction results of the synthesized mesoporous TiO_2_ and the mesoporous TiO_2_ incorporated with extracts of *N. variegata*. From Figure 1a, it can be observed that the synthesized mesoporous TiO_2_ corresponds to the anatase crystalline phase, as evidenced by the main diffraction peaks associated with the reflection planes (101), (103), (004), (112), (200), (105), (211), (213), (204), (116), (220), (215), and (301). This X-ray diffraction (XRD) pattern involving mesoporous TiO_2_ has also been identified in other studies available in the literature.^36,37^ Moreover, the diffraction peaks are well-defined, indicating the presence of a material with an orderly parallel arrangement and good crystallinity.

**Figure 1:**
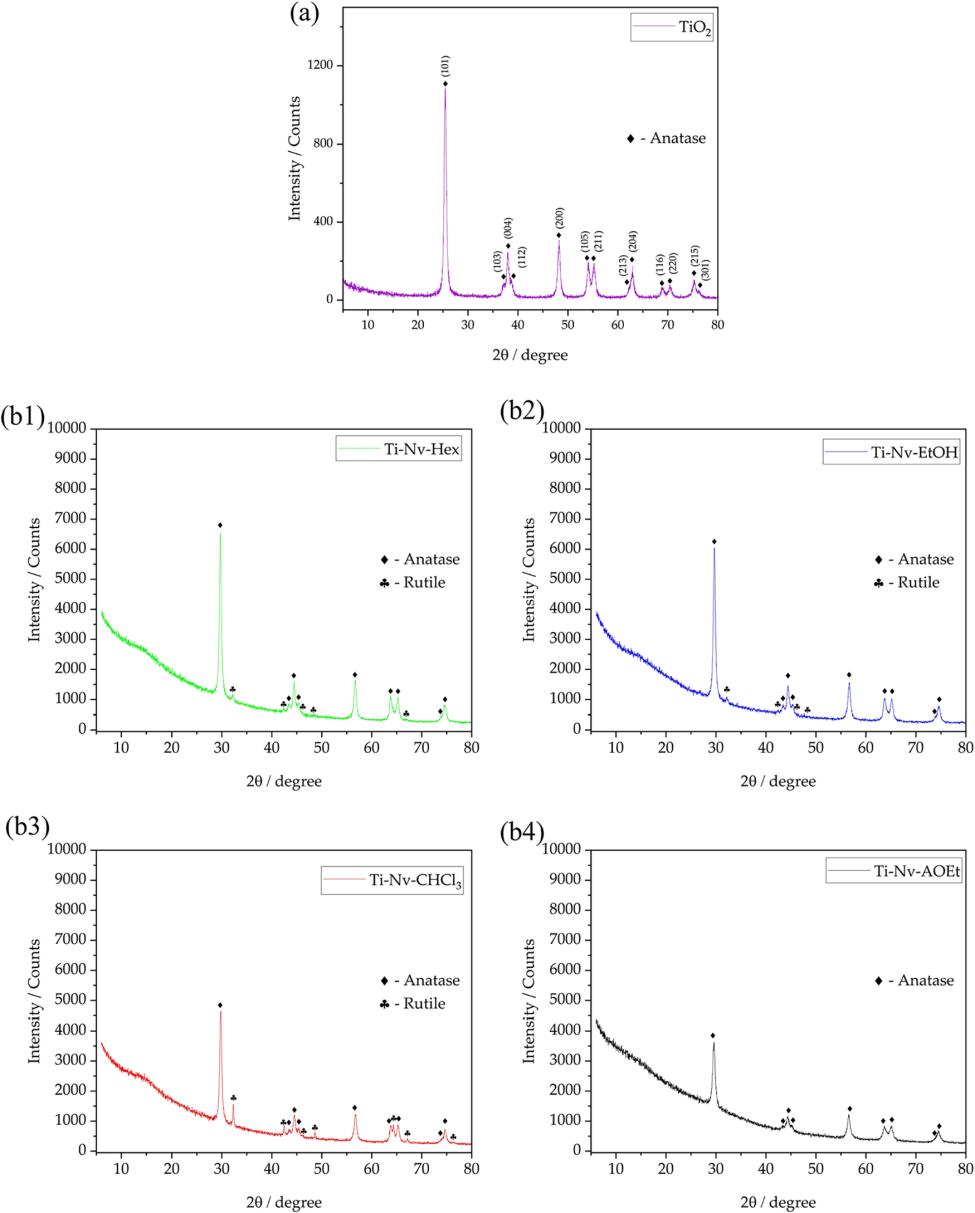
Diffractograms of mesoporous TiO_2_ (a) and mesoporous TiO_2_ with incorporated extracts of *N. variegata* (b).

Figure 1b shows the X-ray diffraction patterns of mesoporous TiO_2_ after the incorporation of *N. variegata* extracts obtained in hexane, ethanol, chloroform, and acetate phases. The introduction of these extracts results in a change in the intensity of the diffraction peaks characteristic of TiO_2_, which suggests the effective incorporation of organic molecules into the channels of the mesoporous material. Additionally, as indicated in Table 2, the characteristic crystallographic planes of TiO_2_ show a shift to higher 2θ angles in the materials with extracts. This modification is interpreted as an indication that the incorporation of the extracts both in the pores and on the surface of the titanium dioxide resulted in an increase in X-ray scattering, which is reflected in the observed change in the diffraction angles. The persistence of the typical titania diffraction peaks in all diffractograms confirms that the crystalline structure of the anatase phase of TiO_2_ was preserved, even after modification with the extracts. This result is crucial, as it demonstrates that the incorporation process does not compromise the structural integrity of the base material, which is an important factor for maintaining its properties.^38,39^

**Table 2:** 2θ Values for the Anatase Phase of TiO_2_ and *N. variegata* Extracts Incorporated into Mesoporous TiO_2_

		anatase												
Material	plane	(101)	(103)	(004)	(112)	(200)	(105)	(211)	(213)	(204)	(116)	(220)	(215)	(301)
TiO_2_I CDD no. 01–089–4921	2θ	25.52°	37.02°	38.00°	38.66°	48.30°	54.06°	55.20°	62.27	63.03°	68.81°	70.40°	75.23°	76.25°
Ti-Nv-Hex ICDD no. 01–073–1764		29.77°	43.42°	44.47°	45.38°	56.65°	63.78°	65.19°	73.80°	74.46°				
Ti-Nv-EtOH ICDD no. 01–073–1764		29.67°	43.47°	44.41°	45.38°	56.65°	63.66°	65.15°	73.78°	74.48°				
Ti-Nv-CHCl_3_ICDD no. 01–073–1764		29.76°	43.49°	44.48°	45.40°	56.75°	63.79°	65.21°	73.82°	74.61°				
Ti-Nv-AcOEt ICDD no. 01–073–1764		29.65°	43.37°	44.29°	45.22°	56.60°	63.69°	65.11°	73.76°	74.47°				

As observed in the XRD patterns of Figure 1b and the data in Table 3, the Ti-Nv-Hex, Ti-Nv-EtOH, and Ti-Nv-CHCl_3_ materials showed the appearance of the rutile phase of TiO_2_ after the incorporation of the extracts. This resulted in materials with a mixture of the anatase and rutile phases. The occurrence may be related to the distinct chemical composition of these extracts, which were incorporated into the pores and onto the surface of the mesoporous TiO_2_. Similar results are reported in the literature.^39–41^

**Table 3:** 2θ Values for the Rutile Phase of *N. variegata* Extracts Incorporated into Mesoporous TiO_2_

		rutile						
material	plane	(110)	(101)	(200)	(111)	(211)	(220)	(310)
TiO_2_	2θ							
Ti-Nv-Hex ICDD no. 01–089–0554		32.22°	42.35°	45.86°	48.38°		66.92°	
Ti-Nv-EtOH ICDD no. 01–089–4920		32.21°	42.28°	45.94°	48.39°		67.03°	
Ti-Nv-CHCl_3_ ICDD no. 01–078–1510		32.27°	42.43°	45.99°	48.61°	64.33°	67.11°	76.28°
Ti-Nv-AcOEt								

The micrographs of the mesoporous TiO_2_ samples and mesoporous TiO_2_ with incorporated *N. variegata* extracts are shown in Figure 2. According to Figure 2a1, the pure mesoporous TiO_2_ exhibits microparticles with a well-defined spherical shape and smooth surface, showing a homogeneous appearance. These microparticles have an average size of 1.6 μm. In the other micrographs (Figure 2a2–a5), it can be observed that the incorporation of *N. variegata* extracts caused significant changes in the morphology and size of the particles. The samples with incorporated extracts exhibited structures with rougher surfaces and irregular contours, indicating the formation of aggregates and possible surface coating of TiO_2_ by the plant extract compounds. Additionally, there is a noticeable increase in the average particle size, reaching approximately 10 μm, along with greater polydispersity compared to the nonfunctionalized material. These changes clearly demonstrate that the incorporation of the extracts into the mesoporous TiO_2_ directly impacts the structural organization of the material, promoting the formation of microparticles with more complex morphological features.^42,43^

**Figure 2:**
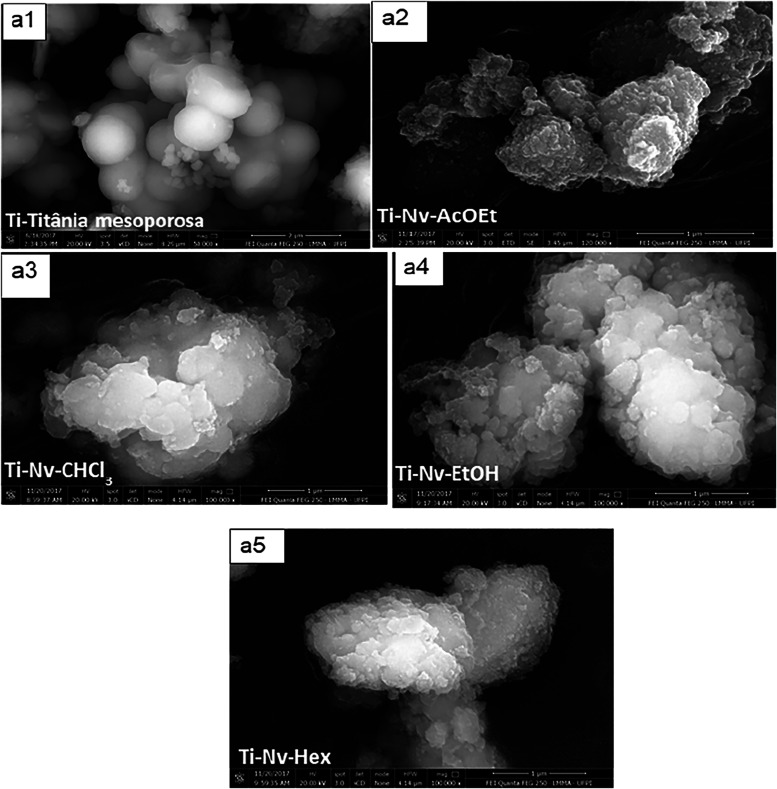
SEM images of mesoporous TiO_2_ (a1) and mesoporous TiO_2_ with *N. variegata* extract incorporated into the acetate Ti-Nv-AcOEt (a2), chloroform Ti-Nv-CHCl_3_ (a3), ethanolic Ti-Nv-EtOH (a4) and hexane Ti-Nv-Hex (a5) phases.

Figure 3 shows the nitrogen (N_2_) adsorption/desorption isotherm results of pure mesoporous TiO_2_ and mesoporous TiO_2_ with incorporated *N. variegata* extracts. According to the figure, pure mesoporous TiO_2_ shows the highest nitrogen adsorption capacity, reaching volumes above 300 cm^3^ g^–1^ at relative pressures close to 1.0. The isotherm can be classified as Type IV, typical of mesoporous materials, and it exhibits a well-defined H1-type hysteresis loop at a *P*/*P*_0_ ratio >0.75. This behavior is characteristic of materials with uniform and well-organized cylindrical pores. Such a combination of Type IV isotherm and H1 hysteresis is generally associated with structures composed of regular channels or compact aggregates of uniform spheres, indicating high porosity, efficient capillary condensation, and a large specific surface area.^44–46^

**Figure 3:**
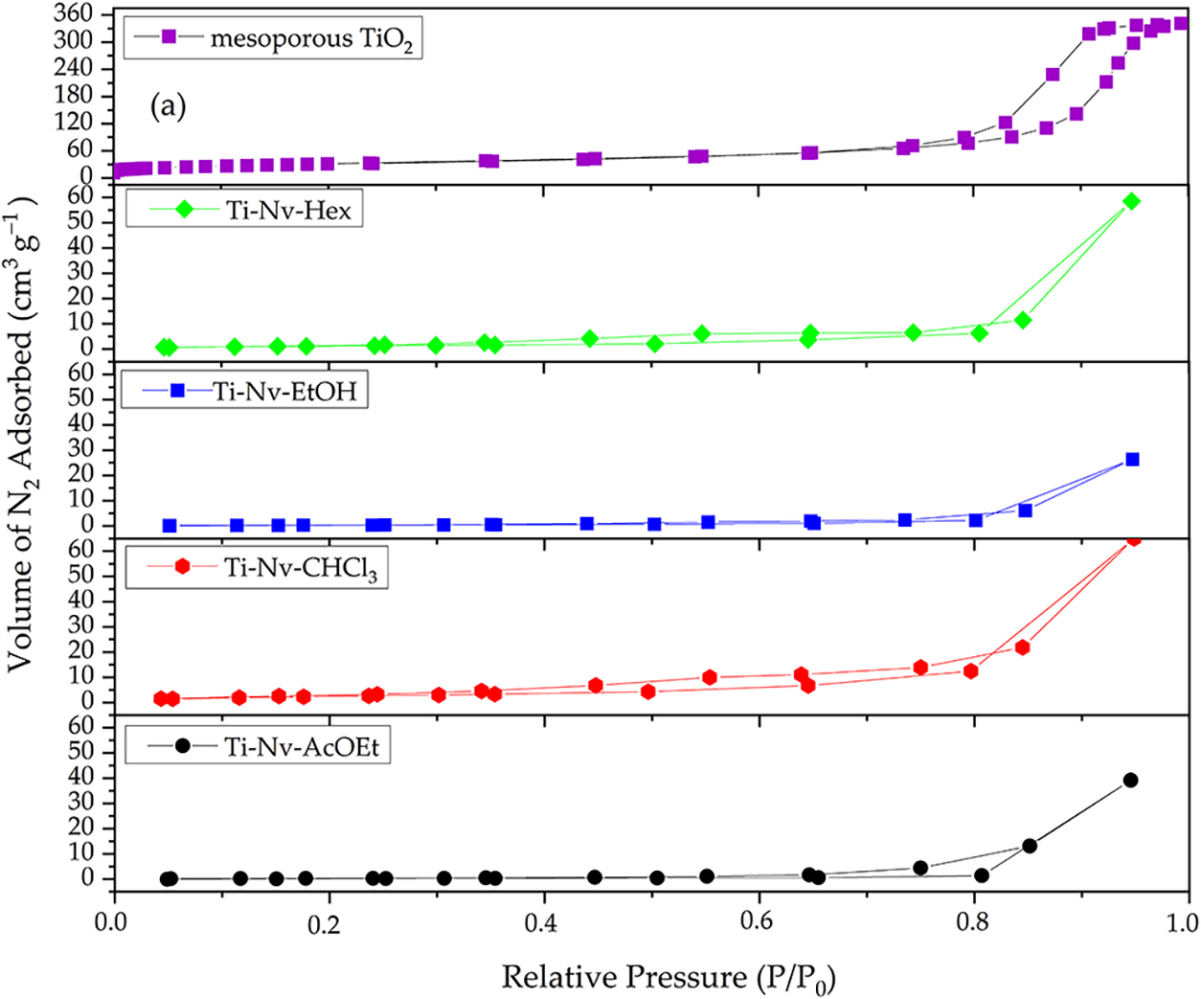
Sorption/desorption isotherms of nitrogen for mesoporous TiO_2_ and mesoporous TiO_2_ with incorporated *N. variegata* extracts.

In the materials incorporated with *N. variegata* extracts, a significant reduction in the volume of adsorbed nitrogen is observed, indicating partial occupation of the pore channels by the plant extract compounds, thus confirming the effectiveness of the incorporation. The adsorption/desorption isotherms of all modified samples can be classified as type IV, characteristic of mesoporous materials; however, they exhibit a distinct hysteresis behavior compared to pure TiO_2_. The curves exhibit H3-type hysteresis loops, noticeable by their steeper and more asymmetrical shape, especially in regions of high relative pressure (*P*/*P*_0_ > 0.8). This type of hysteresis is associated with slit-shaped pores, typically formed by lamellar aggregates or particles with a flat morphology, and indicates a disordered porous structure with a heterogeneous pore size distribution. Unlike pure TiO_2_, which displays an H1-type hysteresis, associated with uniform cylindrical pores, the materials containing extracts do not show clear saturation plateaus, even at high pressures.^45–48^

This change in the hysteresis profile results from the reduction in the average pore diameter, caused by the presence of plant extracts adsorbed within the channels of the mesoporous TiO_2_. This partial pore blockage leads to a decrease in the total volume of adsorbed gas and alters the relative pressure range (*P*/*P*_0_) at which capillary condensation occurs.^45,46^

Figure 4 shows the infrared spectra (FTIR) of pure mesoporous TiO_2_, bioactive extracts of *N. variegata* and pure mesoporous TiO_2_ with incorporated extracts of *N. variegata*. According to Figure 4a, the spectrum of pure mesoporous TiO_2_ exhibits two well-defined bands in the regions of 3500 and 526 cm^–1^. These bands correspond, respectively, to the vibrational stretching of hydroxyl groups (Ti–OH) and the stretching vibrations of Ti–O bonds.^49,50^

**Figure 4:**
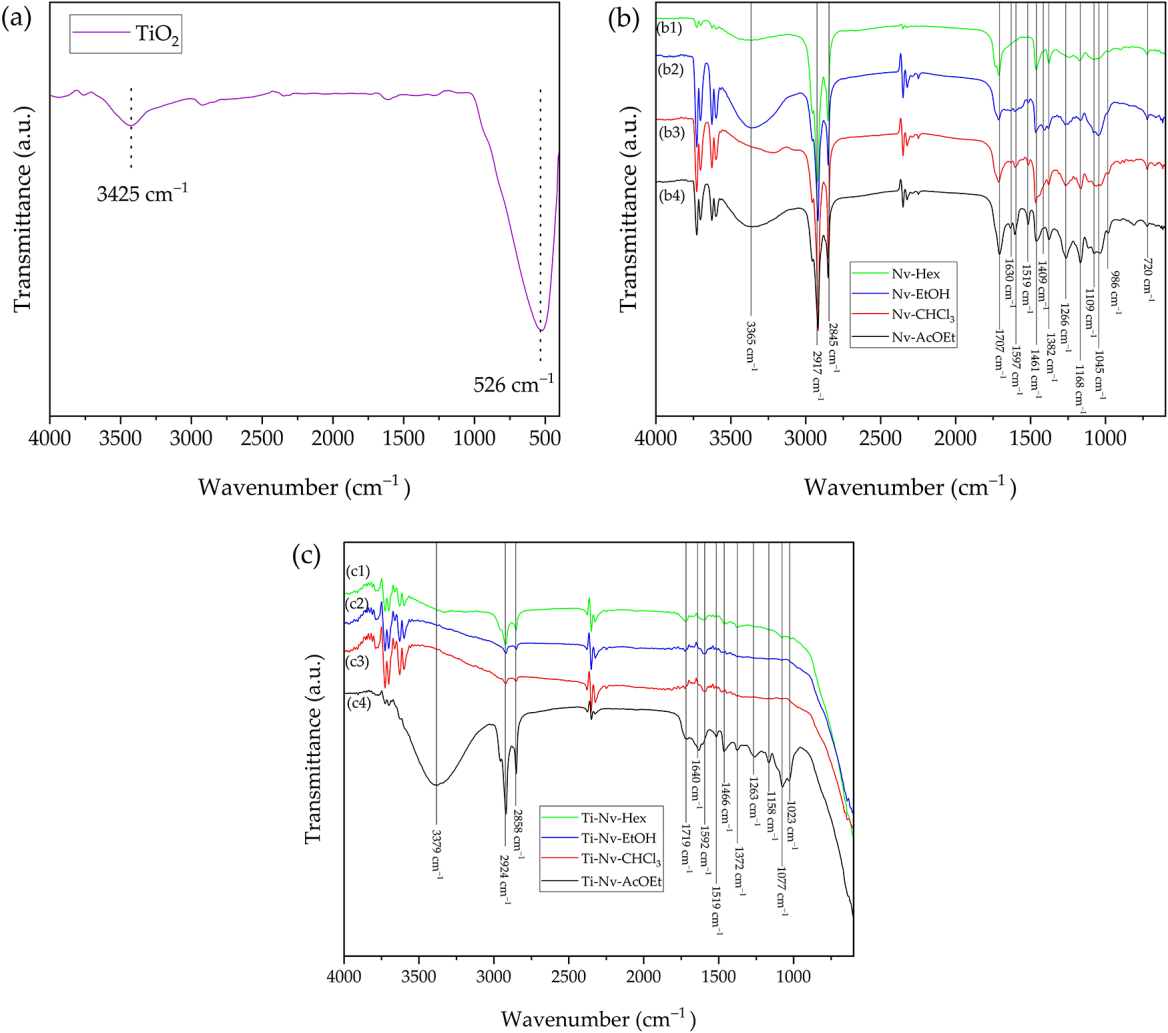
FTIR spectra of pure mesoporous TiO_2_ (a), bioactive extracts of *N. variegata* (b) and pure mesoporous TiO_2_ with incorporated extracts of *N. variegata* (c).

Figure 4b shows the infrared spectra obtained for the *N. variegata* extracts in different solvents (Nv-AcOEt, Nv-CHCl_3_, Nv-EtOH, and Nv-Hex). The presence of common bands is observed, along with other features that vary according to the polarity of the solvent used. The common bands at 2917 and 2845 cm^–1^ are associated with the symmetric and asymmetric stretching of the C–H bond in alkyl groups. The band at 1707 cm^–1^ is attributed to the carbonyl (C=O) stretching, while the band at 1461 cm^–1^ corresponds to the stretching of C=C bonds in aliphatic chains or aromatic rings. The angular deformation of the methyl group (CH_3_) is observed at 1382 cm^–1^. The bands at 1168, 986, and 720 cm^–1^ indicate C–O stretching and ring deformations associated with acids and esters present in all phases.^51–54^ Among the distinct bands, the regions at 1597 and 1519 cm^–1^, present only in the Nv-AcOEt, Nv-CHCl_3_, and Nv-EtOH phases, stand out and are attributed to the C=C stretching of aromatic rings, indicating the presence of phenolic compounds. The band at 3365 cm^–1^, observed in the Nv-AcOEt and Nv-EtOH phases, is related to the hydroxyl group (O–H) of alcohols and phenols. In contrast, the Nv-Hex phase is dominated by bands associated with aliphatic compounds, such as fatty acids, as evidenced by the absence of aromatic bands.^54,55^

Figure 4c reveals that the incorporation of *N. variegata* extracts into mesoporous TiO_2_ caused significant changes in the infrared spectra, distinguishing them from both the pure extracts and the isolated inorganic material. An important observation is the significant reduction in the intensity of the band at 1707 cm^–1^. This band is associated with the stretching vibration of the carbonyl bond (C=O), and its decrease suggests that hydrogen bonds were established between the C=O groups of the organic compounds in the extract and the Ti–OH groups present on the surface of the TiO_2_. Variations in the intensity of the bands above 3300 cm^–1^ were observed, indicating changes in the O–H stretching region. These alterations are associated with the formation of hydrogen bonds between the hydroxyl groups of the functional groups in the extracts and the hydroxyl groups on the surface of the TiO_2_. This supports the existence of intermolecular interactions that reinforce the integration between the organic and inorganic phases. Additionally, the band at 3379 cm^–1^ showed an increase in intensity for the mesoporous TiO_2_-incorporated AcOEt extract sample (Ti-Nv-AcOEt) compared to the pure extract (Nv-AcOEt). This suggests the formation of stronger intermolecular hydrogen bonds between the OH groups of the extract and the OH groups of the TiO_2_, leading to greater integration between the two materials.^56^

Figure 5 shows the thermogravimetric analysis (TGA) curves of the materials studied, where significant differences can be observed between pure TiO_2_ and the mesoporous TiO_2_ systems incorporated with *N. variegata* extracts. Pure TiO_2_ exhibited two main mass loss events: the first, occurring between 323 and approximately 523 K, was related to the removal of physically adsorbed water; and the second, at temperatures above 823 K, was attributed to the condensation of Ti–OH groups forming Ti–O–Ti linkages with the release of water vapor. These results are consistent with the literature regarding the thermal behavior of mesoporous TiO_2_ materials.^57,58^

**Figure 5:**
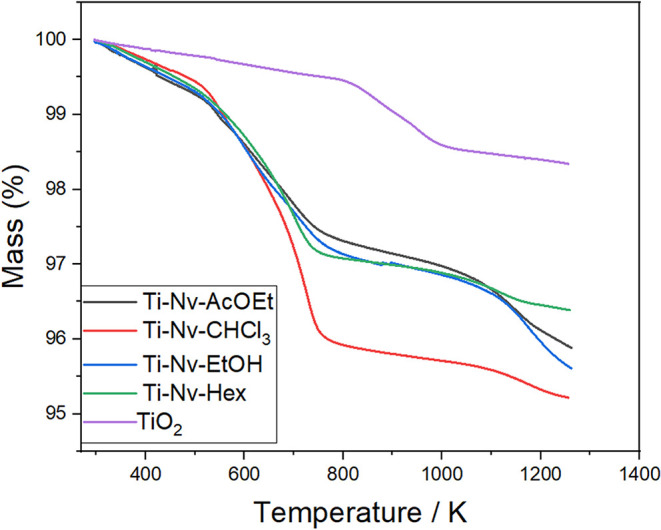
Thermogravimetric curves (TGA) of pure mesoporous TiO_2_ and pure mesoporous TiO_2_ with incorporated extracts of *N. variegata*.

The materials Ti-Nv-AcOEt, Ti-Nv-CHCl_3_, Ti-Nv-EtOH, and Ti-Nv-Hex exhibited distinct thermal degradation profiles compared to pure TiO_2_. Three well-defined mass loss events were identified in the thermogravimetric curves. The first event, observed between 323 and 503 K, corresponded to an average mass loss of 0.7% across all systems, attributed to the removal of physically adsorbed water. The second and more pronounced event occurred between 643 and 773 K and was associated with the thermal decomposition of organic compounds derived from the incorporated *N. variegata* extracts. The mass losses recorded in this interval were 2.15% for Ti-Nv-AcOEt, 2.25% for Ti-Nv-EtOH, 2.35% for Ti-Nv-Hex, and 3.42% for Ti-Nv-CHCl_3_. This variation in mass loss indicates that the extracts incorporated into the mesoporous TiO_2_ possess different thermal stability according to their chemical composition. The type of substances present in each extract directly influences the material’s degradation profile. The third event, observed above 973 K, was attributed to the condensation of Ti–OH groups, leading to the formation of Ti–O–Ti bonds and resulting in an approximate mass loss of 0.80%.^59,60^

Based on the TGA data, the amount of *N. variegata* extract incorporated into the mesoporous TiO_2_ matrices was estimated to be 0.65% for Ti-Nv-AcOEt, 0.75% for Ti-Nv-EtOH, 0.85% for Ti-Nv-Hex, and 1.92% for Ti-Nv-CHCl_3_. These values are consistent with the elemental carbon analysis, which indicated carbon contents of 1.70%, 1.71%, 2.00%, and 2.06% for Ti-Nv-CHCl_3_, Ti-Nv-AcOEt, Ti-Nv-Hex, and Ti-Nv-EtOH, respectively. Overall, the results of the thermogravimetric analysis and elemental analysis confirm the effective incorporation of *N. variegata* extracts into the mesoporous TiO_2_ matrices, although in low percentages (<2.10%), while preserving the crystalline structure and thermal properties of the material.

In TGA, the Ti-CHCl_3_ sample with incorporated CHCl_3_ showed greater mass loss, due to the fact that the main compound identified was palmitic acid, as shown by the mass spectrum (MS) for (*m*/*z* 255.3 [M – H]^−^). Since palmitic acid has a much smaller structural formula when compared to the structures of compounds such as dicaffeoylglycerides and oleic acid. Therefore, due to palmitic acid having a smaller structural formula and the presence of its carboxyl (−COOH) functional group, these characteristics provided greater chemical interaction with the Ti–OH and Ti–O–Ti functional groups of mesoporous TiO_2_.

### Determination of Total Phenolics Content and Antioxidant Activity

The results of total phenolic content quantification, determined by the Folin-Ciocalteu method, are presented in Table 4. A significant variation was observed among the different fractions extracted from *N. variegata*: the ethanolic extract (Nv-EtOH) showed the highest total phenolic content (1.550 ± 0.041 mg GAE g^–1^), followed by the ethyl acetate extract (Nv-AcOEt), with 1.346 ± 0.004 mg GAE g^–1^. The chloroform (Nv-CHCl_3_) and hexane (Nv-Hex) extracts exhibited lower values, 0.900 ± 0.125 and 0.845 ± 0.060 mg GAE g^–1^, respectively. These data confirm the presence of phenolic compounds in all the analyzed fractions, a pattern consistent with results reported in the literature.^8–10,61^

**Table 4:** Quantification of Phenols in the Bioactive Extracts of *N. variegata*

sample	total phenolics content (mg GAE g^–1^)	DPPH (IC_50_, μg mL^–1^)
Nv-AcOEt	1.346 ± 0.004	59.34 ± 1.44
Nv-CHCl_3_	0.900 ± 0.125	125.30 ± 1.54
Nv-EtOH	1.550 ± 0.041	49.03 ± 1.24
Nv-Hex	0.845 ± 0.060	1054.06 ± 5.83
quercetin		8.93 ± 1.16

The results of the antioxidant activity of *N. variegata* extracts, evaluated using the DPPH radical scavenging method, are presented in Table 2. It can be observed that the ethanolic (Nv-EtOH) and ethyl acetate (Nv-AcOEt) fractions showed the best antioxidant performance, with IC_50_ values of 49.03 ± 1.24 and 59.34 ± 1.44 μg mL^–1^, respectively. In contrast, the chloroform (Nv-CHCl_3_) and hexane (Nv-Hex) fractions exhibited lower antioxidant activity, with IC_50_ values of 125.30 ± 1.54 and 1054.06 ± 5.83 μg mL^–1^, respectively. These results demonstrate a direct correlation between the phenolic compound content and the antioxidant efficiency of the extracts, as the fractions with higher total phenolic content also showed lower IC_50_ values, indicating a greater capacity to neutralize free radicals. This pattern is consistent with results reported in the literature.^9,62^

### Mass Spectrometry

Figure 6 displays the mass spectra (MS) of the *N. variegata* extracts, which allows for the identification of the main components in each fraction. In the ethyl acetate fraction (Nv-AcOEt), the signal obtained by ESI-IT-MS in negative mode, with an *m*/*z* of 415.20 ([M – H]^−^), is attributed to dicaffeoylglycerides. These are phenolic metabolites found in nature and known for their significant antioxidant activity. Similarly, the ethanolic fraction (Nv-EtOH) showed a main peak at *m*/*z* 415.3, also corresponding to dicaffeoylglycerides, confirming the presence of bioactive phenolic compounds in both fractions. In the chloroform fraction (Nv-CHCl_3_), the major compound identified was palmitic acid (*m*/*z* 255.3 [M – H]^−^), a naturally occurring saturated fatty acid with low antioxidant potential. Finally, in the hexane fraction (Nv-Hex), the predominant compound was oleic acid (*m*/*z* 281.4 [M – H]^−^), a monounsaturated fatty acid with well-known health benefits, but with lower effectiveness as an antioxidant.^63–66^

**Figure 6:**
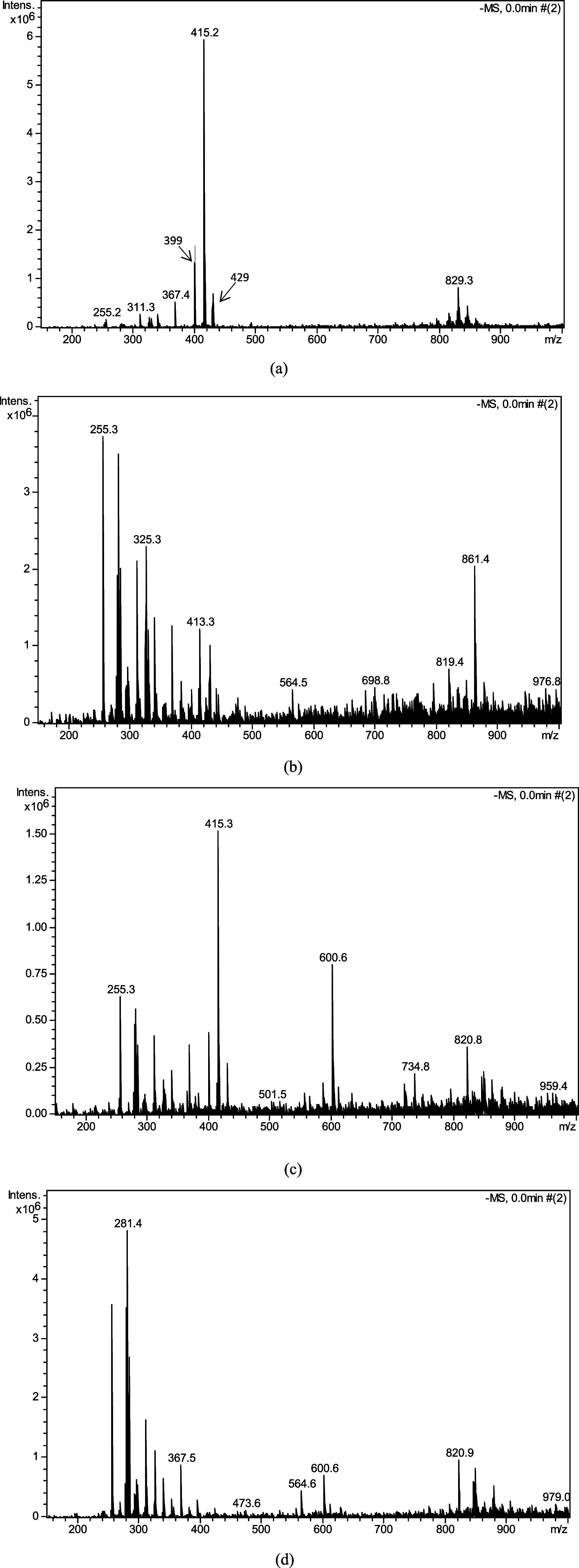
Mass spectra (MS) of the *N. variegata* extracts: Nv-AcOEt (a), Nv-CHCl_3_ (b), Nv-EtOH (c), and Nv-Hex (d).

Mass spectrometry (MS) analysis (Figure 6) revealed that, in addition to dicaffeoylglycerides, other phenolic compounds were identified in the Nv-EtOH and Nv-AcOEt extracts, but not in the Nv-Hex and Nv-CHCl_3_ extracts. For example, the Nv-EtOH extract contained compounds such as feruloyl-caffeoylglycerol (*m*/*z* 429), *p*-coumaroyl-caffeoylglycerol (*m*/*z* 399), and rutin (*m*/*z* 609). The Nv-AcOEt extract, in turn, contained coumaric acid (*m*/*z* 163), caffeic acid (*m*/*z* 179), ferulic acid (*m*/*z* 193), quercetin (*m*/*z* 301), and rutin (*m*/*z* 609). This distinction in chemical composition is directly responsible for the observed differences in total phenolic content and antioxidant activity. The predominance of these phenolic compounds, especially the flavonoids quercetin and rutin, in the Nv-AcOEt and Nv-EtOH fractions, correlates with the higher phenolic content and lower IC_50_ values. In contrast, the Nv-CHCl_3_ and Nv-Hex fractions, which are primarily composed of fatty acids, showed lower phenolic content and reduced antioxidant activity. This reinforces the strong correlation between the chemical composition and the bioactivity of the extracts. Some compounds identified in the mass spectra of *N. variegata* extracts are shown in Table 5.

**Table 5:** Compounds Identified in the Mass Spectra of *N. variegata* Extracts

compound	ESI [M–H]^−^	MS^2^	refs
dicaffeoylglycerol dimer	829		^63–65^
rutin	609	575(31), 563(35), 463(5), 415(28), 353(23), 343(8), 301(100)	^64^
feruloyl-caffeoylglycerol	429	253(100), 235(42), 193(63), 161(49), 135(35)	^63^
dicaffeoylglycerides	415	253(100), 179(11), 161(33), 135(12)	^63^
*p*-coumaroyl-feruloylglycerol	413	393(22), 365(100), 349(9), 249(5), 235(71), 193(21), 177(35), 161(25), 135(9)	^63^
*p*-coumaroyl-caffeoylglycerol	399	253(100), 235(25), 163(21), 145(9), 135(9)	^63^
galacturonide derivative	367	177(100)	^65^
quercetin	301	286(49), 283(100), 272(13), 259(30), 257(38), 187(28), 179(25), 171(24), 158(23), 151(31)	^64^
oleic acid	281		^64^
palmitic acid	255		^66^
ferulic acid	193	178(14), 161(5), 149(63), 134(100)	^63^
caffeic acid	179	161(1), 151(2), 135(100)	^63^
coumaric acid	163	119(100)	^63^

### Toxicity in Blood Agar

The results of the hemolytic activity on 2% sheep blood agar (Table 6) demonstrate that, among the bioactive extracts of *N. variegata* (ethyl acetate, chloroform, ethanolic, and hexane), only the ethyl acetate fraction (Nv-AcOEt) exhibited slight hemolysis in one of the replicates, with the formation of a halo approximately 8 mm in diameter. Similarly, the Nv-AcOEt sample incorporated with TiO_2_ also showed slight hemolysis (−/+), indicating that the presence of the oxide did not prevent the hemolytic effect observed in this fraction. The other samples, including the isolated extracts and their formulations with TiO_2_, showed no hemolytic activity and were therefore considered hemocompatible.

**Table 6:** Hemolytic Activity in Blood Agar^a^

concentrations of 1000 μg mL^–1^						
repetition/extract	1	2	3	4	positive control (polaxamer)	negative control (solvents)
chloroform extract	–/–	–/–	–/–	–/–	+/+	–/–
acetate extract	–/–	–/–	–/–	–/+	+/+	–/–
ethanolic extract	–/–	–/–	–/–	–/–	+/+	–/–
hexane extract	–/–	–/–	–/–	–/–	+/+	–/–
TiO_2_	–/–	–/–	–/–	–/–	+/+	–/–
chloroform extract/TiO_2_	–/–	–/–	–/–	–/–	+/+	–/–
acetate extract/TiO_2_	–/–	–/–	–/–	–/+	+/+	–/–
ethanolic extract/TiO_2_	–/–	–/–	–/–	–/–	+/+	–/–
hexane extract/TiO_2_	–/–	–/–	–/–	–/–	+/+	–/–

a (−/−): no hemolysis; *­(−/+): slight hemolysis; ***­(+/+): hemolysis >1 cm.

The discrete halo formation indicates a low cytotoxic interaction with erythrocytes, suggesting that the compounds present in these specific fractions may induce slight cell lysis. However, since the response was limited and did not occur in all replicates, the hemolytic potential can be considered restricted and dependent on the specific chemical composition of the acetate fraction. The ethanolic, chloroform, and hexane extracts, both in their pure form and combined with TiO_2_, did not induce any level of hemolysis, further supporting their biocompatibility. These results are in agreement with literature reports that have described the hemocompatibility of systems containing TiO_2_.^67–69^

### Toxicity against *Artemia salina*

The Figure 7b presents the toxicity assessment of *N. variegata* extracts in the Nv-AcOEt (ethyl acetate), Nv-CHCl_3_ (chloroform), Nv-EtOH (ethanolic), and Nv-Hex (hexane) fractions after 24 h of exposure. It is observed that the Nv-AcOEt fraction exhibited low toxicity at concentrations ranging from 10 to 250 μg mL^–1^, with survival rates exceeding 90%. However, at higher concentrations (500 and 1000 μg mL^–1^), the viability of *A. salina* was significantly reduced to 33%, indicating a dose-dependent toxic effect. The chloroform fraction (Nv-CHCl_3_) also demonstrated low toxicity at the initial concentrations, with 93% survival at 10 μg mL^–1^ and 67% at 100 μg mL^–1^. However, viability progressively decreased from 250 μg mL^–1^ onward, reaching only 27% at the highest concentration tested. These data suggest a moderate and gradual toxicity profile for this fraction. In contrast, the ethanolic extract (Nv-EtOH) showed low toxicity only at the lowest concentrations (10 and 100 μg mL^–1^, with 97% viability). From 250 μg mL^–1^ onward, however, pronounced cytotoxicity was observed, with survival rates below 7%, reaching just 3% at 1000 μg mL^–1^. The hexane fraction (Nv-Hex), in turn, was the most toxic of all, presenting 63% viability even at 10 μg mL^–1^ and reaching critical levels from 100 μg mL^–1^ onward, with only 3% of *Artemia* surviving at the highest concentration.

**Figure 7:**
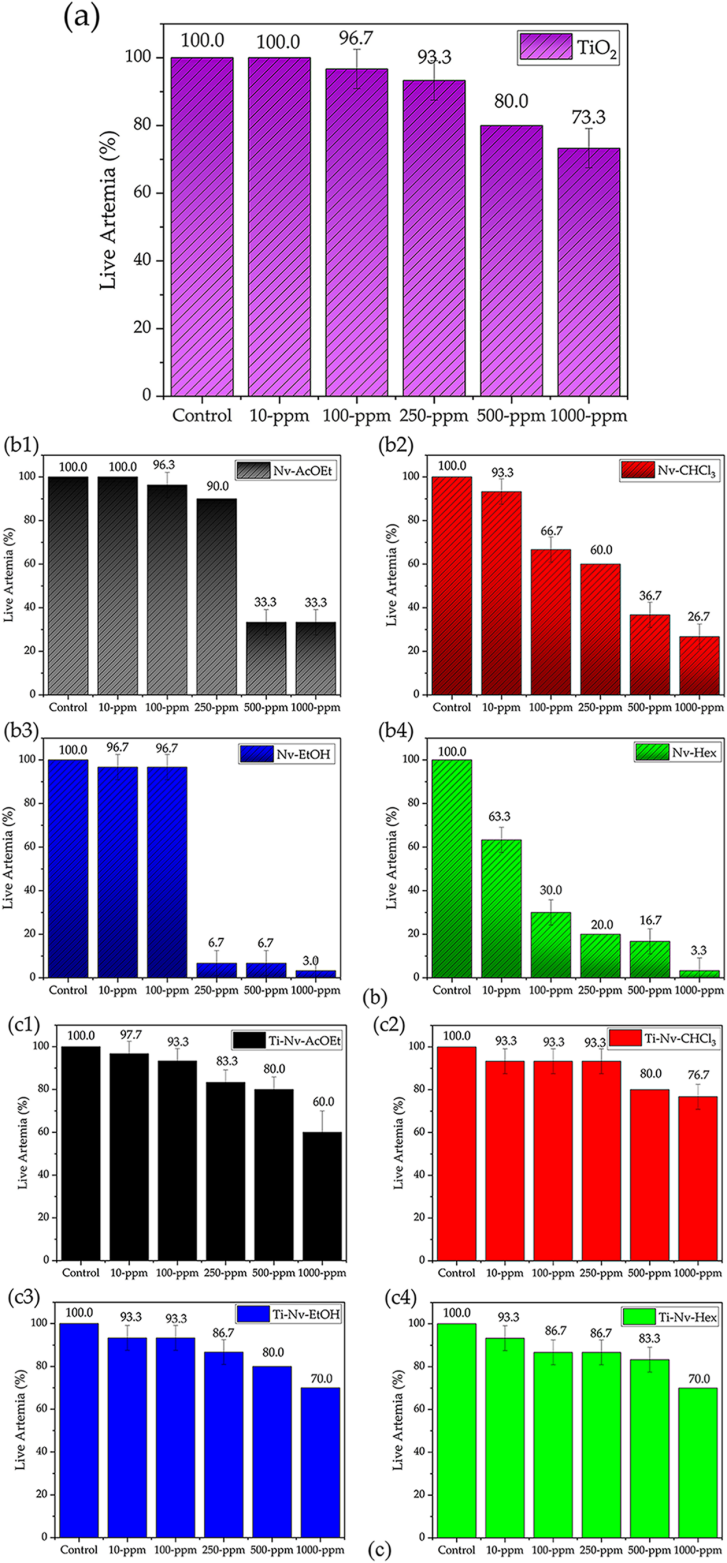
Toxicity to *A. salina* for pure TiO_2_ (a) and for the bioactive extracts of *N. variegata* pure (b) and associated with TiO_2_ (c).

According to Figure 7a, it can be observed that the exposure of *A. salina* nauplii to pure mesoporous TiO_2_ resulted in low toxicity, even at high concentrations. At 10 and 100 ppm, the survival rate remained virtually unchanged compared to the control (100%), with viability of 100% and 96.7%, respectively. From 250 ppm onward, a slight reduction in survival was detected (93.3%), becoming more pronounced at concentrations of 500 ppm (80%) and 1000 ppm (73.3%).

The toxicity results against *A. salina* for the *N. variegata* extracts incorporated into mesoporous TiO_2_ (Ti-Nv-AcOEt, Ti-Nv-CHCl_3_, Ti-Nv-EtOH, and Ti-Nv-Hex) are presented in Figure 7c. The incorporation of the extracts into the porous channels of TiO_2_ resulted in a considerable reduction in the toxicity observed in the pure extracts, suggesting that TiO_2_ acted as a modulator of the release or biological activity of the compounds present in the extracts.

When comparing the data for the ethanolic fractions, the association with TiO_2_ led to a substantial reduction in mortality: at the concentration of 1000 ppm, the survival rate increased from 3% (Nv-EtOH) to 70% (Ti-Nv-EtOH). Similarly, at concentrations of 250 and 500 ppm, viability rates increased from 6.7% to 86.7% and 80%, respectively. Similar results were observed for the hexane fraction, where mortality at 1000 ppm decreased from 96.7% (Nv-Hex) to 30% (Ti-Nv-Hex), highlighting the significant protection provided by mesoporous TiO_2_.The ethyl acetate (Ti-Nv-AcOEt) and chloroform (Ti-Nv-CHCl_3_) fractions also showed improvements in biocompatibility profiles, with survival rates above 60% even at the highest concentrations. These results are consistent with previous studies indicating the low toxicity of mesoporous TiO_2_ within certain concentration ranges, reinforcing its potential as a safe platform for the delivery of bioactive compounds.^70,71^

### pH Evaluation of Sunscreen Formulations

The pH values of sunscreen formulations based on mesoporous TiO_2_ incorporated with *N. variegata* extracts, as well as the commercial standard formulation, were evaluated at 298 K. The mean pH values were as follows: P–Ti–Nv-AcOEt (6.57 ±  0.01), P–Ti–Nv-EtOH (6.72  ± 0.07), P–Ti–Nv-CHCl_3_ (6.57 ±  0.05), P–Ti–Nv-Hex (6.73  ± 0.03), and the standard formulation (6.69  ±  0.03). These values, ranging from 6.57 to 6.73, are very close to the pH of the commercial formulation and fall within the range considered safe and compatible with human skin. Moreover, the pH stability indicates that the incorporation of plant extracts did not compromise the dermatological compatibility of the formulations.^19^

### Determination of Spreadability

The Figure 8 presents the spreadability index (*E_i_*) of the sunscreen formulations, expressed in square millimeters (mm^2^), as a function of the applied mass. All formulations showed a progressive increase in spreadability values with the increase in applied load, ranging approximately from 300 to 5500 mm^2^. This behavior indicates good dispersion capability on the application surface, which is essential to ensure uniform coverage and effective protection against UV rays. Although the spreadability profiles of the different formulations are generally similar, it was observed that the Ti-Nv-AcOEt formulation showed the lowest spreadability indices at all tested masses, suggesting higher viscosity or lower surface mobility. In contrast, the Ti-Nv-Hex formulation presented the highest values, closely followed by Ti-Nv-CHCl_3_. These results indicate that the type of solvent phase used in the incorporation of the extracts can directly influence the rheological behavior of the formulations. According to guidelines for cosmetic formulations, products containing sunscreens must exhibit good spreadability to ensure ease of application and effective performance of the Sun Protection Factor (SPF) on the skin. The observed results, with a consistent increase in the spreading area proportional to the applied mass, indicate that the formulations meet this criterion, maintaining adequate application Properties.^19,34,35^

**Figure 8:**
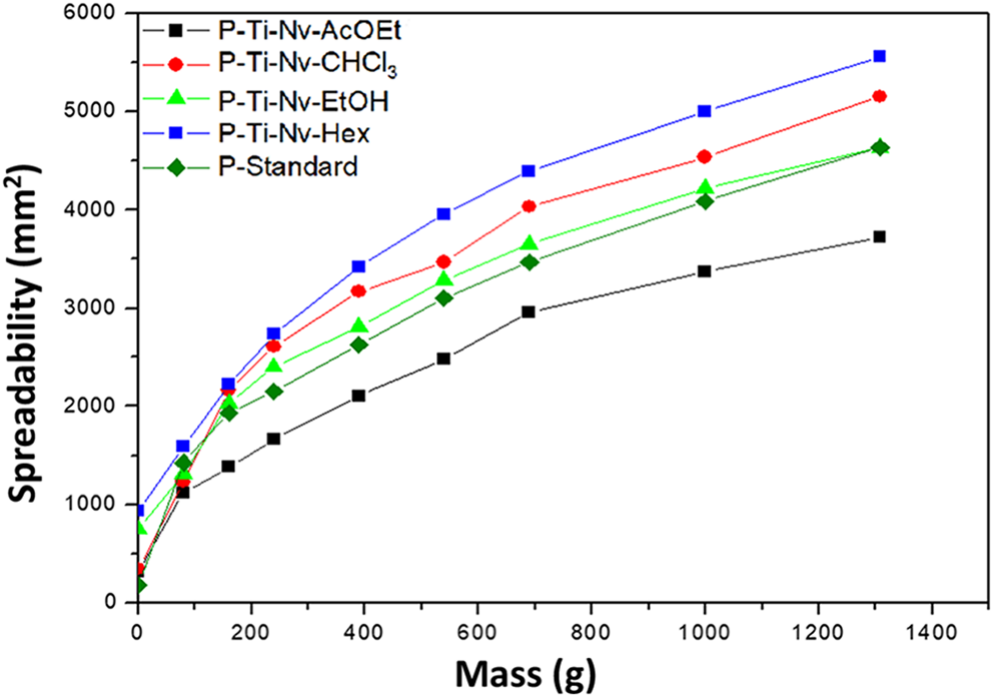
Spreadability of sunscreen formulations based on mesoporous TiO_2_ incorporated with extracts of *Neoglaziovia variegata*.

### Sun Protection Factor (SPF) Test

Figure 9a shows the Sun Protection Factor (SPF) values of *N. variegata* extracts obtained using different solvents: ethyl acetate (Nv-AcOEt), chloroform (Nv-CHCl_3_), ethanol (Nv-EtOH), and hexane (Nv-Hex). It can be observed that the Nv-AcOEt and Nv-EtOH extracts exhibited the highest SPF values, reaching 26 and 10.5, respectively, at a concentration of 100 mg L^–1^. These results are directly related to the higher concentration of phenolic compounds and the antioxidant activity of these fractions, as shown in Table 2. The presence of metabolites with antioxidant properties enhances UV radiation absorption and the neutralization of free radicals generated by sun exposure, contributing to greater photoprotective efficacy. In contrast, the Nv-CHCl_3_ and Nv-Hex extracts, which have lower phenolic content and reduced antioxidant activity, exhibited lower SPF values (3.9 and 1.1, respectively), reinforcing the correlation between phytochemical composition and the photoprotective performance of the extracts.^8–10^

**Figure 9:**
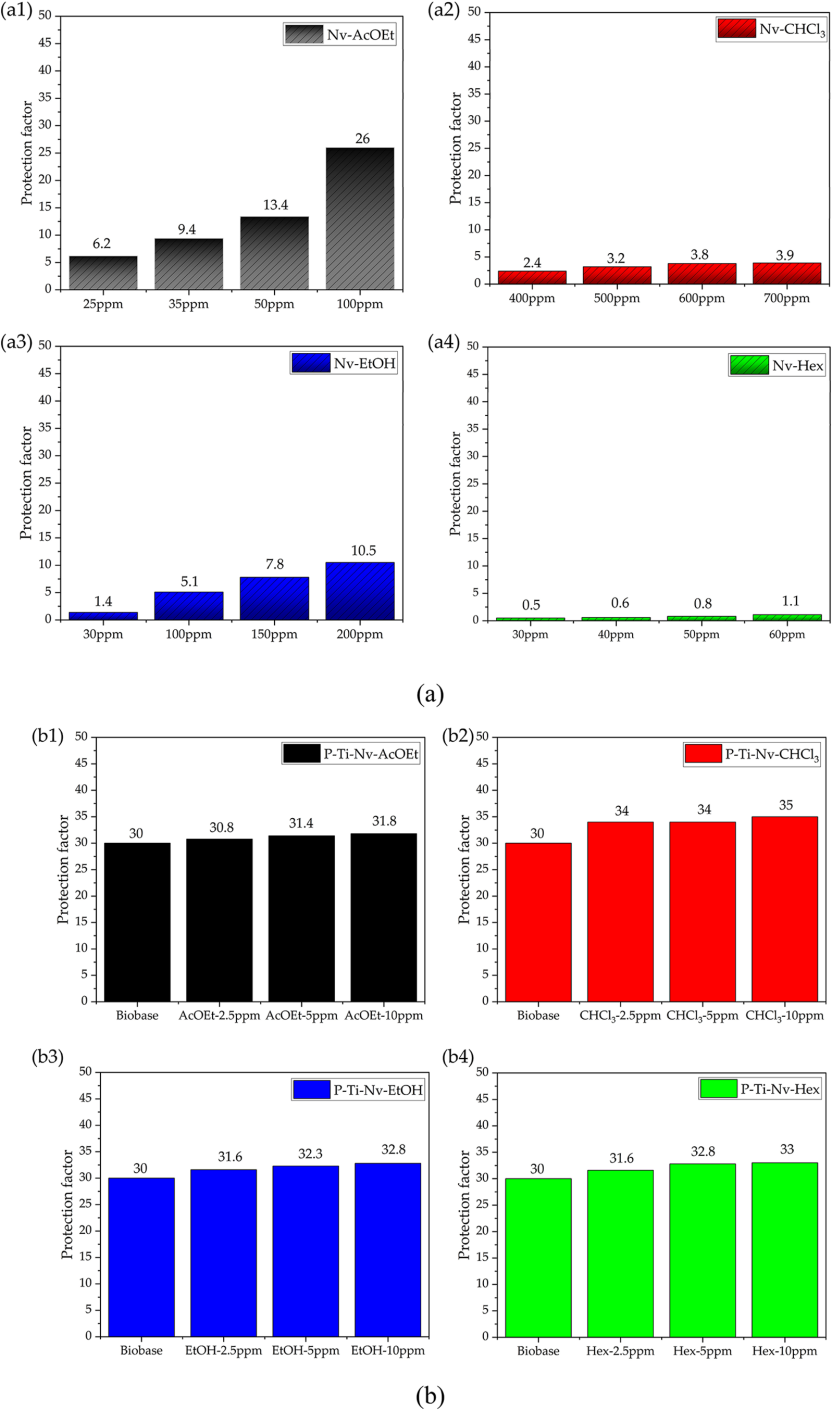
Sun protection factor (SPF) data for pure bioactive extracts of *N. variegata* (a) and those incorporated into mesoporous TiO_2_ (b).

Figure 9b shows the SPF values of the sunscreens developed by incorporating *N. variegata* extracts into mesoporous TiO_2_ dispersed in a commercial cosmetic base, resulting in the formulations P–Ti–Nv-AcOEt, P–Ti–Nv-CHCl_3_, P–Ti–Nv-EtOH, and P–Ti–Nv-Hex. A rising trend in SPF is observed with increasing extract concentrations, even at low ppm levels. Furthermore, when comparing the pure extracts with their respective TiO_2_-containing formulations, a significant enhancement in photoprotective activity is noted. For example, the Nv-CHCl_3_ and Nv-Hex extracts, which initially showed SPF values of 3.9 and 1.1, reached 35 and 33, respectively, after incorporation into TiO_2_. These results highlight the synergistic effect between mesoporous TiO_2_ and the plant’s bioactive compounds.^19,72^

Therefore, the data demonstrate that the incorporation of *N. variegata* extracts into mesoporous TiO_2_ not only enhances the photoprotective activity but also enables the use of low extract concentrations with high efficacy. Thus, these materials represent a promising approach for the development of natural and effective sunscreen formulations.

It is essential to emphasize that further research is needed to precisely identify the compounds in *N. variegata* extracts that provide photoprotection when incorporated into mesoporous TiO_2_. Moreover, investigating how these substances interact with human skin in sunscreen formulations is crucial to ensure application safety and prevent hypersensitivity reactions in users. The promising results of this study open a significant field of research, suggesting that the potential of these materials can be further explored through additional studies, both in the field of sunscreen development and in other biological and human applications.

## Conclusions

This study demonstrated that the incorporation of *N. variegata* extracts from different solvent phases (ethyl acetate, ethanolic, chloroform, and hexane) into mesoporous TiO_2_ was effective, resulting in systems with enhanced photoprotective activity compared to the pure extracts. Structural characterization confirmed the efficient incorporation of the extracts into the porous channels of TiO_2_ without compromising its crystalline structure. Toxicological assays, performed on blood agar and *A. salina*, showed that the developed systems exhibit low toxicity and good biocompatibility, with a significant reduction in adverse effects observed in the pure extracts. Spreadability tests indicated that all formulations presented good coverage capacity, an essential property to ensure uniform application on the skin and, consequently, increased effectiveness of the final product. The Sun Protection Factor (SPF) values showed a significant increase after the incorporation of the extracts into mesoporous TiO_2_, even at low concentrations, highlighting a synergistic effect between the inorganic matrix and the plant bioactive compounds. Thus, the hybrid materials developed represent a promising approach for cosmetic photoprotective formulations, with potential for safe and effective use as an alternative to conventional synthetic UV filters.

## References

[1] Milutinov J., Pavlović N., Ćirin D., Krstonošić M. A., Krstonošić V. (2024). The Potential of Natural Compounds
in UV Protection Products. Molecules.

[2] Yadav, H. K. S. ; Kasina, S. ; Raizaday, A. Chapter 9 Sunscreens. In Nanobiomaterials in Galenic Formulations and Cosmetics; Grumezescu, A. M. , Ed.; William Andrew Publishing: Norwich, NY, USA, 2016; pp 201–230.

[3] Yeager D. G., Lim H. W. (2019). What’s New
in Photoprotection: A Review of New
Concepts and Controversies. Dermatol. Clin..

[4] Carvalho P. G., Naveira R. I., Granone L. I., Mendive C. B., Massa A. E., Churio M. S. (2023). A Comparative Review
of Natural and Synthetic UV Filters:
Gadusol and Benzophenone-3 as Representative Examples. Environ. Adv..

[5] Fonseca M., Rehman M., Soares R., Fonte P. (2023). The Impact
of Flavonoid-Loaded
Nanoparticles in the UV Protection and Safety Profile of Topical Sunscreens. Biomolecules.

[6] Jesus A., Sousa E., Cruz M. T., Cidade H., Lobo J. M. S., Almeida I. F. (2022). UV Filters: Challenges and Prospects. Pharmaceuticals.

[7] Li L., Chong L., Huang T., Ma Y., Li Y., Ding H. (2023). Natural Products and Extracts from Plants as Natural UV Filters for
Sunscreens: A Review. Anim. Models Exp. Med..

[8] Oliveira-Júnior R. G. d., Souza G. R., Ferraz C. A. A., Oliveira A. P., Araújo C. S., Lima-Saraiva S. R. G., Reis S. A. G. B., Gonçalves T. M., Rolim L. A., Rolim-Neto P. J., César F. C. S., Almeida J. R. G. S. (2017). Development and Evaluation of Photoprotective
O/W Emulsions Containing Hydroalcoholic Extract of Neoglaziovia variegata
(Bromeliaceae). Sci. World J..

[9] Oliveira-Junior R. G., Araújo C. S., Souza G. R., Guimarães A. L., Oliveira A. P., Lima-Saraiva S. R. G., Morais A. C. S., Santos J. S. R., Almeida J. R. G. S. (2013). In Vitro Antioxidant and Photoprotective
Activities of Dried Extracts from *Neoglaziovia variegata* (Bromeliaceae). J. Appl. Pharm. Sci..

[10] Gomes M. A., Magalhães B. E.
A., Santos W. N. L., Almeida J. R. G. S. (2022). Influence
of Seasonality on Phytochemical Composition, Phenolic Content and
Antioxidant Activity of *Neoglaziovia variegata* (Bromeliaceae). Biointerface Res. Appl. Chem..

[11] Martínez-Hernández J., Parra-Reyes N., Galindres-Jiménez D. M., Murillo-Acevedo Y., Moreno-Piraján J. C. (2024). Kinetic Study of Phenol, 4-Nitrophenol
and 2,4-Dinitrophenol Photodegradation Using Degussa P25 TiO_2_ and Mesoporous TiO_2_. J. Water Process
Eng..

[12] Motamedisade A., Heydari A., Yin Y., Alotabi A. S., Andersson G. G. (2024). Enhanced
Photocatalytic Degradation of Methyl Orange Using Nitrogen-Functionalized
Mesoporous TiO_2_ Decorated with Au_9_ Nanoclusters. Solar RRL..

[13] Torres-Santos P. T., Farias I. F., Almeida M. D., Passos G. S., Ribeiro L. A. A., Rolim L. A., Pontes M. C., Almeida J. R. G. S., Horta M. C. (2021). Acaricidal Efficacy and Chemical Study of Hexane Extracts
of the Leaves of *Neoglaziovia variegata* (Bromeliaceae) against the Tick Rhipicephalus microplus. Exp. Appl. Acarol..

[14] Peixoto R. D. M., Silva W. E. L., Almeida J. R. G. S., Branco A., Costa M. M. (2016). Antibacterial
Potential of Native Plants from the Caatinga Biome against Staphylococcus
spp. Isolates from Small Ruminants with Mastitis. Rev. Caatinga..

[15] Dantas A. C. S., Machado D. M. R., Araujo A. C., Oliveira-Junior R. G., Lima-Saraiva S. R. G., Ribeiro L. A. A., Almeida J. R. G. S., Horta M. C. (2015). Acaricidal Activity of Extracts from the Leaves and
Aerial Parts of *Neoglaziovia variegata* (Bromeliaceae) on the Cattle Tick Rhipicephalus (Boophilus) microplus. Res. Vet. Sci..

[16] Zi S. C., Chandren S., Yuan L. S., Razali R., Ho C. S., Hartanto D., Mahlia T. M. I., Nur H. (2016). New Method
to Synthesize
Mesoporous Titania by Photodegradation of Surfactant Template. Solid State Sci..

[17] Sounderarajan S., Seenivasan H., Chellam P. V., Puchalapalli D. S. R., Ayothiraman S. (2024). Selective
recovery of esterase from Trichoderma harzianum
through adsorption: Insights on enzymatic catalysis, adsorption isotherms
and kinetics. Int. J. Biol. Macromol..

[18] Pachpawar N. G., Mahajan U. N., Kharwade R. S. (2018). Formulation
and evaluation of sun
protective topical preparation. Int. Res. J.
Pharm..

[19] Rohmani S., Pangesti T. B. (2024). Sunscreen cream formulation from a combination of propolis
extract and titanium dioxide. J. Adv. Pharm.
Educ. Res..

[20] Bora N. S., Mazumder B., Patowary P., Kishor S., Bhutia Y. D., Chattopadhyay P., Dwivedi S. K. (2019). Formulation development and accelerated
stability testing of a novel sunscreen cream for ultraviolet radiation
protection in high altitude areas. Drug Dev.
Ind. Pharm..

[21] Kalegari M., Miguel M. D., Dias J. F. G., Lordello A. L. L., Lima C. P., Miyazaki C. M. S., Zanin S. M. W., Verdam M. C. S., Miguel O. G. (2011). Phytochemical
constituents and preliminary toxicity evaluation of leaves from Rourea
induta Planch. (Connaraceae). Braz. J. Pharm.
Sci..

[22] Mendes A. N., Filgueiras L. A., Siqueira M. R. P., Barbosa G. M., Holandino C., Moreira D. L., Pinto J. C., Nele M. (2017). Encapsulation of *Piper cabralanum* (Piperaceae) nonpolar extract in
poly­(methyl methacrylate) by miniemulsion and evaluation of increase
in the effectiveness of antileukemic activity in K562 cells. Int. J. Nanomed..

[23] Pereira L. A., Reis L. S., Batista F. A., Mendes A. N., Osajima J. A., Silva-Filho E. C. (2019). Biological Properties of Chitosan Derivatives Associated
with the Ceftazidime Drug. Carbohydr. Polym..

[24] Meyer B. N., Ferrigni N. R., Putnam J. E., Jacobsen L. B., Nichols D. E., McLaughlin J. L. (1982). Brine Shrimp:
A Convenient General Bioassay for Active
Plant Constituents. Planta Med..

[25] de
Pinho J. V., Celano M. R., Andrade J., De Almeida A. E. C. C., Hauser-Davis R. A., Conte-Junior C. A., Xing B. (2024). Effects of Salinity on Naphthalene Adsorption and Toxicity of Polyethylene
Microparticles on *Artemia salina*. Chemosphere.

[26] Bonoli M., Verardo V., Marconi E., Caboni M. F. (2004). Antioxidant Phenols
in Barley (*Hordeum vulgare* L.) Flour:
Comparative Spectrophotometric Study among Extraction Methods of Free
and Bound Phenolic Compounds. J. Agric. Food
Chem..

[27] Yahya L. A., Tobiszewski M., Vakh C. (2024). Alkyl Polyglycoside-Assisted Separation
Followed by Smartphone-Based Digital Image Colorimetry for On-Site
Determination of Total Phenolic Content in Plant-Based Milk Alternatives. Microchem. J..

[28] Fraige K., Dametto A. C., Zeraik M. L., Freitas L. de., Saraiva A. C., Medeiros A. I., Castro-Gamboa I., Cavalheiro A. J., Silva D. H. S., Lopes N. P., Bolzani V. S. (2018). Dereplication
by
HPLC-DAD-ESI-MS/MS and Screening for Biological Activities of Byrsonima
Species (Malpighiaceae). Phytochem. Anal..

[29] Gajić I., Dinić A., Stanojević L., Zvezdanović J., Nikolić V., Urošević M., Nikolić L., Savić V. (2024). Osage Orange (*Maclura pomifera* (Raf.) Schneid) Fruit Extracts: UHPLC-DAD-ESI-MS/MS Analysis, Antioxidant
Activity and In Vivo Skin Tests. Nat. Prod.
Res..

[30] Qian Y., Qiu X., Zhu S. (2015). Lignin: A Nature-Inspired Sun Blocker for Broad-Spectrum
Sunscreens. Green Chem..

[31] Darmawan M. A., Ramadhani N. H., Hubeis N. A., Ramadhan M. Y. A., Sahlan M., Abd-Aziz S., Gozan M. (2022). Natural Sunscreen Formulation with
a High Sun Protection Factor (SPF) from Tengkawang Butter and Lignin. Ind. Crops Prod..

[32] Dutra E. A., Oliveira D. A. G. C., Kedor-Hackmann E. R.
M., Santoro M. I. R. M. (2004). Determination
of Sun Protection Factor (SPF) of Sunscreens by Ultraviolet Spectrophotometry. Rev. Bras. Cienc. Farm..

[33] Sayre R. M., Agin P. P., Levee G. J., Marlowe E. (1979). A Comparison of In Vivo
and In Vitro Testing of Sunscreening Formulas. Photochem. Photobiol..

[34] Souza R. P., Holanda J. N. P., Sousa L. R. B., Oliveira D., Souza D. C. P., Sousa R. W. R. (2020). Pharmacotechnical
Development and Quality Control of
a Cryotherapy Gel Based on Ginger Extract, Menthol and Caffeine. Res. Soc. Dev..

[35] Velasco M. F. V., Vieira V. O., Fernandes F. C., Dario R. M., Pinto M. V. B., Kaneko A. R., Baby L. R. (2006). Assessment of SPF of Cosmetic Formulations
Containing UV Filters by Spectrophotometric and HPLC Methods. Braz. J. Pharm. Sci..

[36] Roškarič M., Žerjav G., Zavašnik J., Finšgar M., Pintar A. (2025). Effect of TiO_2_ Morphology on the Properties
and Photocatalytic Activity of g-C_3_N_4_/TiO_2_ Nanocomposites under Visible-Light Illumination. Molecules.

[37] Guo X.-F., Li Q., Ren H.-T., Wang J., Han X. (2025). Homogeneous-Dispersed
Bi–Ag Bimetals over Mesoporous TiO_2_ for Enhanced
Photocatalytic Reduction of Nitrate. Chem. Phys..

[38] Sharafudheen S. B., Vijayakumar C., Anjana P. M., Bindhu M. R., Alharbi N. S., Khaled J. M., Kadaikunnan S., Kakarla R. R., Aminabhavi T. M. (2024). Biogenically
Synthesized Porous TiO_2_ Nanostructures for Advanced Anti-Bacterial,
Electrochemical, and Photocatalytic Applications. J. Environ. Manage..

[39] Ahn E.-Y., Shin S.-W., Kim K., Park Y. (2022). Facile Green Synthesis
of Titanium Dioxide Nanoparticles by Upcycling Mangosteen (Garcinia
mangostana) Pericarp Extract. Nanoscale Res.
Lett..

[40] Anbumani D., Dhandapani K. V., Manoharan J., Babujanarthanam R., Bashir A. K. H., Muthusamy K., Alfarhan A., Kanimozhi K. (2022). Green synthesis
and antimicrobial efficacy of titanium dioxide nanoparticles using
Luffa acutangula leaf extract. J. King Saud
Univ. Sci..

[41] Thakur B. K., Kumar A., Kumar D. (2019). Green synthesis of titanium dioxide
nanoparticles using Azadirachta indica leaf extract and evaluation
of their antibacterial activity. S. Afr. J.
Bot..

[42] Sule R., James U. E. (2024). Synthesis of Mesoporous TiO_2_ Using Aloe
Vera Extract for Solar Cell Applications. Case
Stud. Chem. Environ. Eng..

[43] Haidry A. A., Fatima Q., Wang Z., Wang Y., Ji Y., Raza A. (2023). Optimization of the Specific Surface Area of Ordered
Mesoporous TiO_2_ Yields a High Response to Humidity. Colloids Surf., A.

[44] Carneiro M. T., Morais A.Í.S., de Carvalho Melo A. L. F., Ferreira F. J. L., Santos F. E. P., Viana B. C., Osajima J. A., Bezerra R. D. S., Del
Mar Orta Cuevas M., Peña-Garcia R. R., Almeida L. C., Silva-Filho E. C. (2023). Biochar Derived from Water Hyacinth
Biomass Chemically Activated for Dye Removal in Aqueous Solution. Sustainability.

[45] Groen J. C., Peffer L. A. A., Pérez-Ramírez J. (2003). Pore Size
Determination in Modified Micro- and Mesoporous Materials. Pitfalls
and Limitations in Gas Adsorption Data Analysis. Microporous Mesoporous Mater..

[46] El-Badry B. A., Aldaghri O., Ibnaouf K. H., Younis A. M., Albadri A., Alluhayb A. H., Ben Aissa M. A., Modwi A. (2024). Efficacy of Mesoporous
TiO_2_–ZrO_2_@g-C_3_N_4_ Produced Using a Simple Ultrasonic Approach for Copper Ion Removal
from Wastewater. J. Sci.: Adv. Mater. Devices.

[47] Taufik A., Muzakki A., Saleh R. (2018). Effect of
Nanographene Platelets
on Adsorption and Sonophotocatalytic Performances of TiO_2_/CuO Composite for Removal of Organic Pollutants. Mater. Res. Bull..

[48] Buttersack C., König A., Gläser R. (2019). Stability of a Highly Dealuminated
Y-Zeolite in Liquid Aqueous Media. Microporous
Mesoporous Mater..

[49] Ani I. J., Akpan U. G., Olutoye M. A., Hameed B. H., Egbosiuba T. C. (2024). Adsorption–Photocatalysis
Synergy of Reusable Mesoporous TiO_2_–ZnO for Photocatalytic
Degradation of Doxycycline Antibiotic. Heliyon.

[50] Akpan U. G., Hameed B. H. (2011). Solar Degradation
of an Azo Dye, Acid Red 1,
by Ca–Ce–W–TiO_2_ Composite Catalyst. Chem. Eng. J..

[51] Sun Y., Liu N., Zhao L., Liu Q., Wang S., Sun G., Zhao Y., Zhou D., Cao R. (2024). Attenuated Total Reflectance–Fourier
Transformed Infrared Spectroscopy (ATR-FTIR) Coupled with Deep Learning:
A Rapid Method for Geographical Origin Identification of Sea Cucumber
Apostichopus japonicus. Microchem. J..

[52] Gonçalves A. P.
B., Cruz A. M. F., Sales J. C., Souza M. C., Silva F. L. B. M., Guimarães D. H., Mattedi S., José N. M. (2016). Achievement
and Characterization of Cellulose Nanowhiskers of Palm (*Elaeis guineensis*) and Bromelia Fibers (*Neoglaziovia variegata*). Chem.
Eng. Trans..

[53] Tapia-Maruri D., Evangelista-Lozano L., Alamilla-Beltrán B. H., Camacho-Díaz S. V., Ávila-Reyes J. C., Villalobos-Espinosa A. R., Jiménez-Aparicio A. R. (2022). Comparative Evaluation of the Thermal,
Structural, Chemical and Morphological Properties of Bagasse from
the Leaf and Fruit of Bromelia hemisphaerica Lam. Delignified by Organosolv. Appl. Sci..

[54] Zishan M., Dhar D. W., Manzoor U. (2024). Fourier Transform Infrared Spectroscopy
Analysis and Phytochemical Screening of Selected Medicinal Plant Extracts. Afr. J. Bio. Sci..

[55] d’Almeida J. R. M., d’Almeida A. L. F. S., de Carvalho L. H. (2008). Mechanical,
Morphological, and Structural Characteristics of Caroa (*Neoglaziovia variegata*) Fibres. Polym. Polym. Compos..

[56] Wellia D. V., Syuadi A. F., Rahma R. M., Syafawi A., Habibillah M. R., Arief S., Kurnia K. A., Saepurahman, Kusumawati Y., Saefumillah A. (2024). Rind of Aloe vera (L.) Burm. f Extract for the Synthesis
of Titanium Dioxide Nanoparticles: Properties and Application in Model
Dye Pollutant Degradation. Case Stud. Chem.
Environ. Eng..

[57] Mittal R., Patar S., Sharma A., Bhateria R., Bhardwaj A. K., Kashyap R., Bhukal S. (2025). Surface Modified Novel Synthesis
of Spirulina Assisted Mesoporous TiO_2_@CTAB Nanocomposite
Employed for Efficient Removal of Chromium (VI) in Wastewater. Appl. Surf. Sci..

[58] Hilonga A., Kim J.-K., Sarawade P. B., Kim H. T. (2010). Mesoporous Titania–Silica
Composite from Sodium Silicate and Titanium Oxychloride. Part II:
One-Pot Co-Condensation Method. J. Mater. Sci..

[59] Kumar K. V. S., Bindu M., Suresh S., Anil A., Sujoy S., Mohanan A., Periyat P. (2023). Investigation
on Swelling Behavior
of Sodium Alginate/Black Titania Nanocomposite Hydrogels and Effect
of Synthesis Conditions on Water Uptake. Results
Eng..

[60] Penchev H., Zaharieva K., Dimova S., Tsacheva I., Eneva R., Engibarov S., Lazarkevich I., Paunova-Krasteva T., Shipochka M., Mladenova R., Dimitrov O., Stoyanova D., Stambolova I. (2024). Green Synthesized Composite AB-Polybenzimidazole/TiO_2_ Membranes with Photocatalytic and Antibacterial Activity. Crystals.

[61] Pap N., Marnila P., Pihlava J.-M., Tienaho J. (2024). Optimization of Ultrasound
Assisted Extraction of the Sea Buckthorn Leaves, Characterization
of the Phenolic Compounds, and Determination of Bioactive Properties
of the Extracts. Future Foods..

[62] Abduh M. Y., Nofitasari D., Rahmawati A., Eryanti A. Y., Rosmiati M. (2023). Effects of
Brewing Conditions on Total Phenolic Content, Antioxidant Activity
and Sensory Properties of Cascara. Food Chem.
Adv..

[63] Ma C., Xiao S.-Y., Li Z.-G., Wang W., Du L.-J. (2007). Characterization
of Active Phenolic Components in the Ethanolic Extract of Ananas
comosus L. Leaves Using High-Performance Liquid Chromatography
with Diode Array Detection and Tandem Mass Spectrometry. J. Chromatogr. A.

[64] Hu L., Zhang H., Zhang X., Cheung W. W., Hu Y., Hong A., Guo J., Xu Y., He J., Lu J., Deng H., Zhu Y., Cai Q. (2025). Untargeted Screening
and Differential Analysis of Bioactive Compounds in Male and Female
Silkworm (Bombyx mori) Pupae through Orbitrap Exploris Mass Spectrometry. Food Chem..

[65] Quéméner B., Cabrera
Pino J. C., Ralet M. C., Bonnin E., Thibault J. F. (2003). Assignment
of acetyl groups to O-2 and/or O-3 of pectic oligogalacturonides using
negative electrospray ionization ion trap mass spectrometry. J. Mass Spectrom..

[66] Richter K., Nygren H., Malmberg P., Hagenhoff B. (2007). Localization
of Fatty Acids With Selective Chain Length by Imaging Time-of-Flight
Secondary lon Mass Spectrometry. Microsc. Res.
Technol..

[67] Niranjan R., Kaushik M., Selvi R. T., Prakash J., Venkataprasanna K. S., Prema D., Pannerselvam B., Venkatasubbu G. D. (2019). PVA/SA/TiO_2_-CUR Patch for Enhanced Wound
Healing Application: In Vitro
and In Vivo Analysis. Int. J. Biol. Macromol..

[68] Salimi N., Mohammadi-Manesh E. (2025). Effect of
Graphene Oxide on Fe_3_O_4_/TiO_2_ Nanocomposite
and Their Characterization for Drug
Delivery Applications. Inorg. Chem. Commun..

[69] Gobi R., Babu R. S. (2025). In-Vitro Investigation
of Chitosan/Polyvinyl Alcohol/TiO_2_ Composite Membranes
for Wound Regeneration. Biochem. Biophys. Res.
Commun..

[70] Ramírez-Concepción H. R., Anaya-Esparza L. M., García-Magaña M. L., Yahia E. M., Meza-Espinoza L., Montalvo-González E. (2023). The Effects
of Chitosan-TiO_2_ and Chitosan-TiO_2_-ZnO-MgO Hybrid
Coatings on the Shelf Life of Jackfruit Bulbs (Artocarpus heterophyllus
Lam). Int. J. Food Sci. Technol..

[71] Anaya-Esparza L. M., González-Silva N., Yahia E. M., González-Vargas O. A., Montalvo-González E., Pérez-Larios A. (2019). Effect of
TiO_2_-ZnO-MgO Mixed Oxide on Microbial Growth and Toxicity
against *Artemia salina*. Nanomaterials.

[72] Kanthik T., Lokham S., Sungthongjeen S. (2020). Development
of Sunscreen Products
Containing Titanium Dioxide and Aloe Vera Gel. Key Eng. Mater..

